# Progress in Gelatin as Biomaterial for Tissue Engineering

**DOI:** 10.3390/pharmaceutics14061177

**Published:** 2022-05-31

**Authors:** Izeia Lukin, Itsasne Erezuma, Lidia Maeso, Jon Zarate, Martin Federico Desimone, Taleb H. Al-Tel, Alireza Dolatshahi-Pirouz, Gorka Orive

**Affiliations:** 1 NanoBioCel Research Group, School of Pharmacy, University of the Basque Country (UPV/EHU), 01006 Vitoria-Gasteiz, Spain; izeia.lukin@ehu.eus (I.L.); itsasne.erezuma@ehu.eus (I.E.); lmaeso13@hotmail.com (L.M.); jon.zarate@ehu.eus (J.Z.); 2 Bioaraba, NanoBioCel Research Group, 01009 Vitoria-Gasteiz, Spain; 3 Biomedical Research Networking Centre in Bioengineering, Biomaterials and Nanomedicine (CIBER-BBN), Institute of Health Carlos III, Av Monforte de Lemos 3-5, 28029 Madrid, Spain; 4 Consejo Nacional de Investigaciones Científicas y Técnicas (CONICET), Instituto de Química y Metabolismo del Fármaco (IQUIMEFA), Facultad de Farmacia y Bioquímica Junín 956, Universidad de Buenos Aires, Buenos Aires 1113, Argentina; desimone@ffyb.uba.ar; 5 Sharjah Institute for Medical Research, University of Sharjah, Sharjah 27272, United Arab Emirates; taltal@sharjah.ac.ae; 6 Department of Health Technology, Center for Intestinal Absorption and Transport of Biopharmaceuticals, Technical University of Denmark, 2800 Kgs Lyngby, Denmark; aldo@dtu.dk; 7 University Institute for Regenerative Medicine and Oral Implantology-UIRMI (UPV/EHU-Fundación Eduardo Anitua), 01007 Vitoria-Gasteiz, Spain; 8 Singapore Eye Research Institute, The Academia, 20 College Road, Discovery Tower, Singapore 169856, Singapore

**Keywords:** gelatin, biomaterials, regenerative medicine, tissue engineering

## Abstract

Tissue engineering has become a medical alternative in this society with an ever-increasing lifespan. Advances in the areas of technology and biomaterials have facilitated the use of engineered constructs for medical issues. This review discusses on-going concerns and the latest developments in a widely employed biomaterial in the field of tissue engineering: gelatin. Emerging techniques including 3D bioprinting and gelatin functionalization have demonstrated better mimicking of native tissue by reinforcing gelatin-based systems, among others. This breakthrough facilitates, on the one hand, the manufacturing process when it comes to practicality and cost-effectiveness, which plays a key role in the transition towards clinical application. On the other hand, it can be concluded that gelatin could be considered as one of the promising biomaterials in future trends, in which the focus might be on the detection and diagnosis of diseases rather than treatment.

## 1. Introduction

Tissue engineering is defined as a new branch of knowledge that is a result of combining technologies from different research areas including biology, chemistry, engineering, medicine, pharmacy or material science [1]. This interdisciplinary field can provide a medical alternative in the current health issue of organ and tissue failure. The US government has recently reported that 107,000 people are on the waiting list for organ transplantation and as many as 17 people on these lists die every day [2]. In Europe, every hour, six patients join the waiting list and, approximately, 18 of them die every day [3]. In 2017, 22.3 million bone-related procedures were executed, and by 2022, there is expected to be an increase of 30% [4].

In this line, tissue engineering has gained importance as it relies on designing, developing and optimizing three-dimensional (3D) scaffolds for tissue repair, healing and regeneration [1]. With this important development in hand, tissues with a high donor demand, such as bone and cardiac, have taken advantage of this revolutionary multidisciplinary area and dealt with donor scarcity [1]. In addition, some of the studies have reached clinical trials, being proof of the capacity of designed systems to regenerate tissue [5,6,7]. Nonetheless, although tissue engineering has opened up a new way of practicing medicine, there are some concerns in terms of cost-effectiveness, reproducibility or scalability that need to be addressed [8].

The development of new technologies such as 3D bioprinting led to burgeoning interests in the use of these technologies. On the one hand, this forefront approach permits integration into scaffolds of an array of composites such as biological agents or cells, bringing about enhanced system effectiveness. On the other hand, its unique feature with respect to automation enables the manufacturing of complex scaffolds cost-effectively, together with higher reproducibility rates, hence addressing the lack of clinical translation [4,9]. In fact, it has already been employed in a wide range of tissues including skeletal muscle, bone and neural regeneration [10,11,12].

The latest advancements in 3D bioprinting have paved the way for the new trend known as 4D bioprinting, which has demonstrated promising results so far. This 4D bioprinting allows 3D-printed structures to respond to stimuli such as pH or temperature, in accordance with the changes the tissue may encounter over time [13,14,15]. This new methodology is a great advantage, as it helps to design structures that can better mimic natural tissue, as well as adapt to interfaced tissues [16].

It is worth bearing in mind that these new technologies are used in certain biomaterials. Particularly in the field of tissue engineering, these materials must meet the following requirements: good biocompatibility and biodegradability and low toxicity, among others. Thus, one of the most widely used biomaterials in the area is gelatin. Specifically, the fact that it originates from collagen makes this material suitable for orthopedics and it has already been widely employed in a myriad of systems such as drug delivery systems, hydrogels, scaffolds or films for wound dressing [16,17,18,19]. Additionally, the Food and Drug Administration (FDA) has approved a blend of demineralized bone matrix and gelatin (DBX Strips) for bone tissue engineering, together with absorbable gelatin sponges (Surgiflo^®^, Ferrosan Medical Devices A/S, Søborg, Denmark, Cutanplast^®^, Mascia Brunelli S.p.a., Milano, Italy) to maintain hemostasis in multiple surgeries [20,21].

Due to the aforementioned properties, researchers have continued to use this versatile biomaterial. Hence, this review focuses on the latest progress with gelatin in the field of tissue engineering. Current challenges together with the latest advances and most significant results of gelatin-based approaches are discussed.

## 2. Gelatin as a System

Since the launch of biomaterials for tissue regeneration purposes, gelatin has been used in several systems such as injectable hydrogels, drug delivery systems and scaffolds. This section highlights the advantages and disadvantages of using gelatin as a biomaterial in the field of tissue engineering.

Gelatin is a natural polymer that bears a resemblance to its precursor collagen (one of the most abundant components of the extracellular matrix). As previously described, after isolating collagen, gelatin can be extracted in two ways: by alkaline or acid hydrolysis [22,23,24]. The latter will determine the isoelectric point (IP) of gelatin. When subjected to acid hydrolysis, gelatin is classified as type A with IP ≈ 5. Extraction in an alkaline medium gives rise to type B gelatin with IP ≈ 9. It is worth mentioning that, as a result of the denaturalization step, gelatin has a linear structure, consisting of Gly-X-Y (mainly proline and hydroxyproline) sequences. Similarly, other amino acid sequences in the structure, known as the RGD motif, help in cell adhesion, proliferation and differentiation.

Its advantageous characteristics, mainly biocompatibility, biodegradability and low toxicity, allow for increased cell adhesion, differentiation and proliferation at the same time that it is degraded by body enzymes (metalloproteinases), without causing an immunogenic response [22,25,26]. Moreover, as it is cost-effective, it has been employed in a wide range of tissues (bone, skeletal, neural), forming different systems that range from microparticles for bone regeneration enhancement, to wound dressing or hydrogels for the controlled release of chemotherapeutic agents in the treatment of cancer [27,28,29].

Although its benefits make this material appropriate to be used in tissue engineering, gelatin manipulation also has some potential drawbacks. A noticeable property of this polymer is the lack of thermostability, changing from solid formto gel depending on temperature. One of the most common strategies to overcome this limitation is to crosslink its sequences either physically or chemically [22,24]. Widely described physical methods rely on using UV light or microwave energy to rearrange gelatin’s amino acid sequences, but this approach is more likely to lack efficacy, as it is more difficult to have good control of the crosslink density. There have also been attempts with chemical agents that range from synthetic polymers (glutaraldehyde) to natural enzymes (transglutaminase) [18,30,31,32]. Synthetic polymers have shown controlled synthesis, but the byproducts may result in cytotoxicity, whereas enzymes may not generate chemical waste materials, as they are natural and bond gelatin fibers [30,32,33,34,35]. Nonetheless, this necessity to crosslink gelatin has put aside the idea of forming in situ hydrogels.

On the other hand, gelatin is known for its ability to absorb water. This characteristic is highly valued in tissue regeneration, since porosity ensures a diffusion of nutrients as well as oxygen for proper cell growth [36]. However, porous structures do not always meet all the requirements to provide the exchange of products for cell survival, because either the size or the diameter of the pores is not sufficient, or they are not regular enough, and some gelatin-based cell delivery systems have demonstrated a poor cell survival rate [37]. Along these lines, the current issue lies in finding how to use gelatin-based systems, as a means to ensure proper pore size that may result in a high rate of cell survival.

Finally, gelatin is a polymer that can be obtained from different sources, but the most common is the natural one, for example the porcine skin [16,19,38]. The latter made it the polymer of choice for many researchers. However, the disadvantages stated above, including poor mechanical stability, imply that it has to be combined with other materials to improve its properties. Such a process, in some instances, resulted in complicating the design of the composite system [39]. This, in turn, leads to a lack of reproducibility and reduces cost-effectiveness, and consequently, scalability, leaving behind the translation from in vitro to human use in medical practices.

## 3. New Advances in the Production of Gelatin-Based Constructs

**Figure 1:**
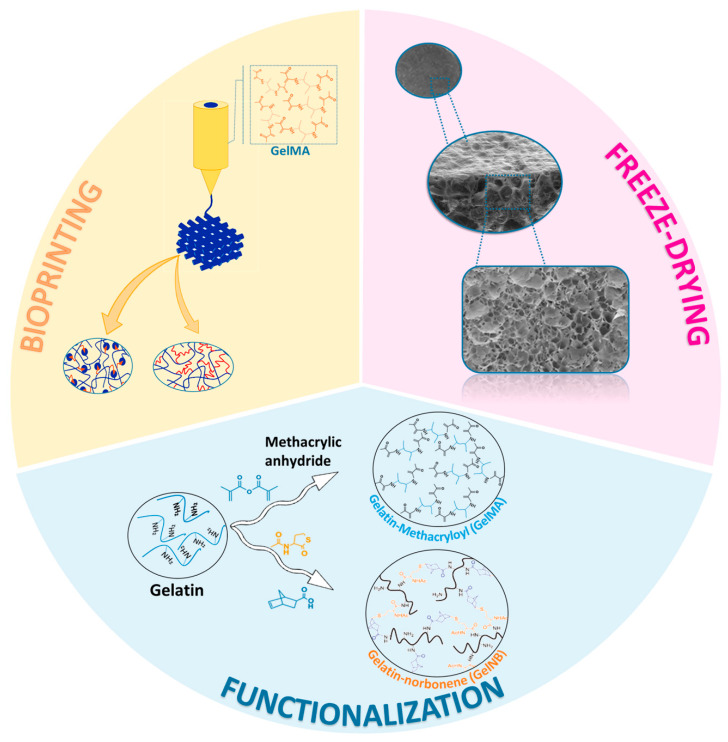
Illustrative image of the latest advances in the design process of gelatin-based systems. Reprinted from International Journal of Pharmaceutics, 562, Echave et al. [30,40]. Enzymatic crosslinked gelatin 3D scaffolds for bone tissue engineering, 151–161, copyright 2019, with permission from Elsevier; Acta Biomaterialia, 94, Tygtal et al. [30,40]. Additive manufacturing of photo-crosslinked gelatin scaffolds for adipose tissue engineering, 340–350, copyright 2019, with permission from Elsevier.

The latest advances in tissue engineering have brought about novel systems that encompass the disadvantages and make gelatin a promising candidate. Owing to that, the pace towards clinical translation might be accelerated. This section describes the progress made in coping with the above-mentioned limitations (Figure 1).

### 3.1. Technological Progress

#### 3.1.1. Bioprinting

High-precision 3D printing permits the design of gelatin-based systems that mimic a tissue-like environment accurately and extends its use in complex applications, such as adipose, blood vessels, skeletal muscle or for wound dressing [28,40,41,42]. For instance, in the search to find an alternative for adipose tissue regeneration, Daikuara et al. designed a simple yet effective gelatin-based hydrogel using the bioprinting technique. This emerging technique enabled the design of adipose tissue by simply varying the gelatin concentration and printing conditions, without the need to combine it with other materials [40].

**Figure 2:**
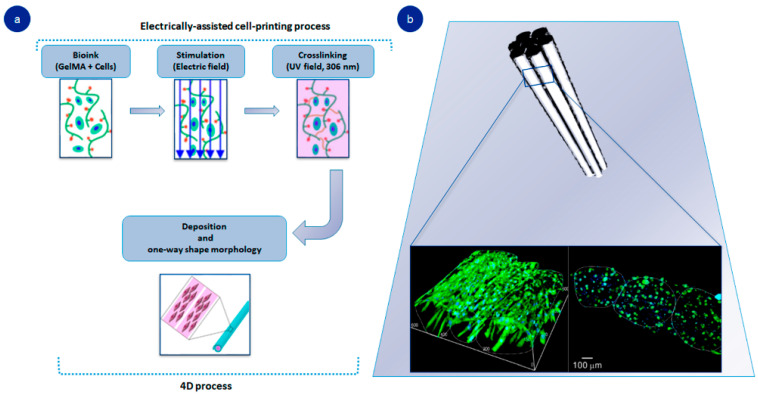
Gelatin-based 4D-printed hydrogel: (**a**) manufacturing process of 4D-printed hydrogel; (**b**) DAPI/MHC cross-sectional images of fibers after 21 days. Adapted with permission from Yang et al. [43]. Theranostics, published by Ivyspring International Publisher, copyright 2021.

In addition, adding the variable of time to 3D bioprinting facilitates the adjustment and application of gelatin directly to the injured site (Figure 2a). An enlightening study described the 4D bioprinting of gelatin by combining a 3D printed gelatin-based hydrogel with electrical stimuli. The system responded to electrical changes, and cells were lined to form fibrous structures that simulated skeletal muscle (Figure 2b). Since this tissue is based on aligned cell constructs, printing allows for inducing electrically engineered hydrogel to rearrange its composition. This method represents a promising development in achieving complex tissues, such as muscle-like enrollment where cell proliferation is linked to electrical stimulation [43]. Within this framework, gelatin is classified as a smart biomaterial, owing to its ability to reshape itself in changing physical conditions such as wettability, or electric or magnetic field. The latter holds great promise in bone tissue engineering applications as it is a stimuli-responsive polymer, which can lead to rigorous drug delivery as well as opening up the opportunity for biosensing and monitoring [44,45]

Another strategy to improve bone tissue regeneration is the incorporation of inorganic molecules within gelatin using different approaches and technologies such as 3D bioprinting [19,46,47]. In this way, it is possible to merge the latest technological advances, which provides homogeneity and accuracy to the system, with gelatin and inorganic molecules present in the mineralized part of the bone [22]. For example, Jeong et al. engineered various scaffolds composed of different gelatin and β-tri-calcium phosphate concentrations. In the study, printing technology demonstrated its accuracy in spreading calcium phosphate nanoparticles uniformly throughout gelatin-based scaffold. Furthermore, scaffolds with the highest amount of gelatin and inorganic molecules allowed for higher bone tissue formation in vivo [19,46].

Printing techniques have also fueled the use of nanoparticles such as nanoclays when using gelatin as a biomaterial. Nanoclays are silicate-derived multilayers that have been shown to play a key role in the physiology of a wide range of tissues such as bone [48]. Several studies have proved that these nanoparticles are able to complement the already adequate properties of gelatin hydrogels for bone tissue regeneration [16,49,50,51]. One of the main advantages of nanoclays consists in improving the gelatin’s poor mechanical properties. In addition, the multilayer structure can facilitate the release of biological agents, and the combination of 3D printing leads to new configurations that better mimic the tissue of origin [52]. As a proof of concept, Quint et al. engineered VEGF-releasing gelatin methacrylol-based scaffolds including embedded laponite nanoparticles [16]. This approach presented several advantages, including the facility of gelatin to crosslink quickly and the use of 3D printers to create blended scaffolds. The addition of nanoclay resulted in a steadier release of the growth factor [16]. These results were in accordance with other studies that demonstrated the great ability of nanoclays to absorb proteins as well as to form tighter hydrogels [49,50,51].

#### 3.1.2. Freeze-Drying Technique

**Figure 3:**
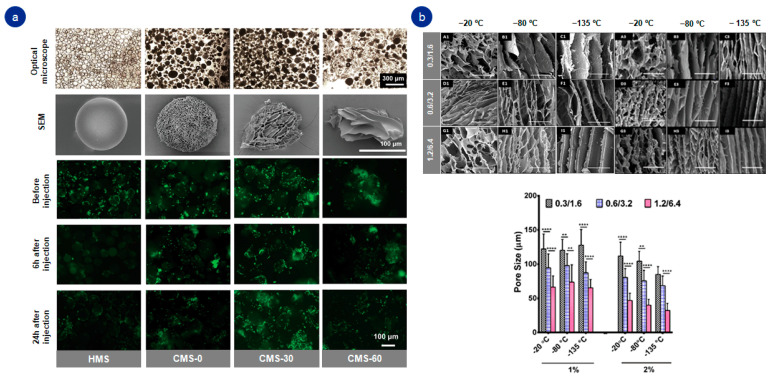
Effect of freeze-drying technique in gelatin-based systems: (**a**) macroscopic and microscopic (SEM) images of freeze-drying effect in gelatin-based hydrogels and viability assay of the cells within gelatin-based hydrogels; (**b**) SEM images and quantitative analysis of the effect of different temperatures and polymer concentration on pore size. ** *p* ≤ 0.01, **** *p* ≤ 0.0001. Adapted with permission from (**a**) Yuan et al. [47]. Small, published by John Wiley and Sons, copyright 2021; (**b**) Singh et al. [12]. Biomacromolecules 2019, 20 (2), 662–673. Copyright 2022, American Chemical Society.

The porous three-dimensional structure of gelatin allows the diffusion of nutrients and oxygen for cell survival and higher cell adhesion rates. However, its porosity is always desirable for regenerative medicine approaches [53]. The freeze-drying technique enables modification of the pore diameter, the result being a reorganized permeable structure capable of improving cell adhesion and the regenerative capacity of gelatin as a biomaterial [18,30]. Therefore, the freeze-drying process could help to produce highly porous and mechanically stable gelatin-based structures (Figure 3).

Recently, Echave et al. developed an enzymatically cross-linked gelatin-based scaffold. The freeze-drying technique enabled gelatin to form a porous hydrogel, which helped to attract cells responsible for osteogenesis [18,30]. In other tissues, such as neural ones, the challenge lies in finding the proper alignment of pores. In that case, temperature cooling helps gelatin to form porous structures and thus better mimic tissue and promote nerve regeneration [12]. Furthermore, this technique has become widespread, as it has also proven to be advantageous for cell protection as well as for the release of biologically active agents [17,36,54,55].

### 3.2. Functionalization

A noteworthy drawback of gelatin is the need to crosslink its chains in order to gain stability performance. This determines the in situ use of this polymer, which is a highly desirable property when treating inaccessible areas [56,57]. One interesting alternative consists of functionalizing and modifying gelatin with the methacryloyl group, which is able to crosslink gelatin amino acids in mild circumstances in terms of temperature and pH [58].

**Figure 4:**
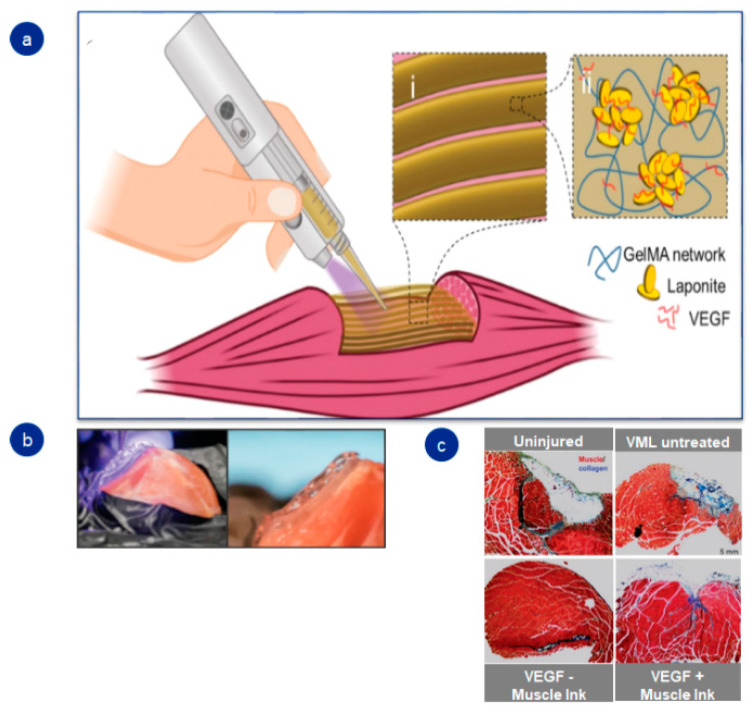
Schematic illustration of 3D portable printer and in vivo outcomes in skeletal muscle: (**a**) graphic diagram; (**b**) images of GelMA-based hydrogel implantation; (**c**) in vivo results of fibrosis in nontreated and treated groups. Adapted with permission from Quint et al. [16]. Advanced Healthcare Materials, published by John Wiley and Sons, copyright 2021.

The chance to merge photosensitive groups with gelatin generates the opportunity to design injectable hydrogels that rapidly crosslink and gelify in the damaged tissues. For example, Quint et al. engineered a transportable printer capable of printing a gelatin-based hydrogel straightaway in the injured skeletal muscle (Figure 4). Combining gelatin with a methacrylol group allowed for photocrosslinking of the hydrogel within seconds once it was injected into the damaged tissue [16].

**Figure 5:**
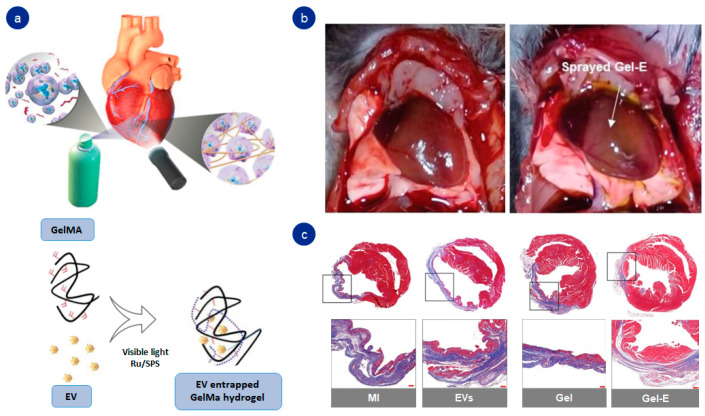
Functionalized gelatin-based hydrogel: (**a**) graphic representation of sprayable hydrogel; (**b**) in vivo implantation of hydrogel into the myocardial tissue; (**c**) trichrome staining results after 28 days of hydrogel implantation. Adapted with permission from Tang et al. [59]. Advanced Healthcare Materials, published by John Wiley and Sons, copyright 2021.

Tang et al. went a step further and designed a sprayable hybrid hydrogel based on methacrylated gelatin. The rapid crosslinking under visible light permitted the placement of the hydrogel directly into the damaged cardiac tissue, with no need to inject it, acting as a network to release extracellular vesicles in a constant manner (Figure 5) [59]. These new systems make it possible to extend the use of gelatin to tissues that are difficult to access and treat, and accelerating the direct administration of the hydrogel into the injured site.

Functionalization of gelatin improves the versatility of the hydrogel and its applicability, but the weak mechanical properties of gelatin-based systems remain a problem. To address this, recent advances have combined methacrylated gelatin (GelMA) hydrogels with nanocomposites. The addition of nanocomposites includes materials that extend conductivity (carbon-based materials, metals) and even minerals such as hydroxyapatite [60]. Lately, an exciting approach to regenerate electrically active tissues (skeletal muscle, neural) based on blending polymer with highly conductive materials has been reported. Xu et al. engineered a conductive system based on GelMA with biodegradable black phosphorous nanomaterial. The electroactive hydrogels showed enhanced ability to differentiate mesenchymal stem cells to neural cells [61].

In the case of bone tissue regeneration, functionalized gelatins have been reinforced with minerals, such as xonotlite (calcium silicate derivative) or nanohidroxyapatite (an abundant material in the inorganic part of bone) [62,63]. The latter allows GelMA hydrogels to improve their mechanical properties and osteoconductivity as well as to approximate the native composition of bone. For example, Li et al. designed and developed nanohydroxyapatite-reinforced GelMA-based hydrogels with enhanced mechanical properties and improved osteogenic ability in guided bone regeneration in vivo [63].

The adding of methacrylol groups has resulted in important progress, but recent studies suggest a new trend in the use thiol-ene photocrosslinked hydrogels [64,65,66]. Unlike gelatin metachrylol, thiol-ene- and norbornene-modified gelatin hydrogels use a lower photoinitiator concentration. Furthermore, norbornen’s high selectivity towards thiols significantly improves cell survival [64]. One interesting example is the norbornene- and thiol-ene-based gelatin system designed by Göckler et al., which demonstrated a fast photocrosslinking process with barely any side reaction, and it enabled a cell survival rate above 80% regardless of the degree of cross-linking. This is particularly important as methacrylate-gelatin-based hydrogels are totally dependent on the level of functionalization [64]. Another interesting approach is the fabrication of active gelatin scaffolds with tailored RGD motifs employing a site-specific enzymatic reaction. This approach facilitated the recruitment of host cells mediated by the specific RGD–integrin interactions and promoted osteogenic differentiation [67].

In summary, functionalization of gelatin may address an important technological and medical need: that is, the rapid use of hydrogels in emergency situations, as it allows the design of systems that can be implemented in a fast and efficient way in the injured site. Nonetheless, much work lies ahead, since most groups that functionalize gelatin have a chemical origin, and thus could lead to undesired side effects.

## 4. State of the Art in Gelatin-Based Systems

Technological advances have introduced numerous gelatin-based systems that range from the ability to regenerate tissue per se to serve as an evaluation-platform for agent delivery. Here, we address the role of gelatin in recent systems that have been designed in the field of tissue regeneration.

### 4.1. Gelatin as Tissue Regenerating Intermediary

One of the purposes of tissue engineering is to create a scaffolding system for cells to provide a suitable environment that ensures tissue repair and healing. In this regard, as gelatin has good biocompatibility, biodegradability and low toxicity, it has been incorporated into some scaffolding systems [68,69]. For instance, Yao and colleagues confirmed the ability of gelatin-based scaffolds to promote osteogenesis both in vivo and in vitro by activating osteoclasts. To that aim, they relied on previously tested, electrically responsive gelatin-based scaffolds. Yet in this study, scaffolds were subjected to an extra electrical stimulus that resulted in osteoclast activation that led to osteoblast differentiation and maturation. In the same tissue, vascularization may play an important role when it comes to regeneration. An elegant study has gone a step further and designed a gelatin-based microspheres scaffold. In vitro results showed the ability to assemble a bone-like endothelial structure, which was subsequently translated as an osteoinductive capacity in vivo. Unlike the vast majority of scaffolds, this forefront macroporous approach could address conventional rigid scaffold limitations, as it is a movable structure with abundant RGD motifs that allows for the formation of capillaries for tissue regeneration [69].

Another strategy that has gained importance recently relies on designing self-healing hydrogels. The latter reveals itself to be an interesting alternative in highly loaded tissues, such as bone, owing to their properties of regeneration after collapse [70,71,72]. In this process, gelatin’s characteristics support formulating hydrogels that are dynamically bonded, which give rise to systems that can be recomposed upon their breakup. Particularly, aromatic molecules of this polypeptide allow for the generation of dynamic bonds, by means of host–guest physical interactions [73]. Likewise, gelatin amino groups permit interaction with aldehyde groups and, therefore, form dynamic bonds [70,71,72]. Based on the latter, Vahedi et al. engineered a gelatin-based self-healing and injectable hydrogel. Specifically, gelatin amino groups were combined with amylopectin aldehyde groups that resulted in hydrogels capable of recovering their shape and maintaining their rheological properties. Along with that, they confirmed their applicability in bone tissue regeneration as scaffolds presenting osteoinductive properties [70].

Much consideration has also been directed towards the development of hydrogels that can adhere to damaged tissues and/or have conductive properties. Since gelatin has the ability to enhance cell adhesion, it may serve as a platform to integrate adhesive hydrogels into the tissue [74]. Similarly, gelatin’s tertiary structure enhances the mechanical properties in adhesive hydrogels [75,76]. As an example, Cao et al. designed an organic hydrogel with high adhesiveness, stretchability and mechanical properties. In that instance, they took advantage of the complex structure of gelatin to crosslink within organic materials in order to improve the mechanical properties of the hydrogel [76].

Despite the fact that gelatin is not a conductive material, it has also been part of conductive hydrogels. As it is a naturally derived material, it confers highly desirable improvements in electrically active elements such as biocompatibility or cell adhesion, which otherwise are lacking in these hydrogels [77,78]. For instance, Hu et al. designed a conductive hydrogel for peripheral nerve reconstruction. Conductivity was achieved by using graphene oxide, while biocompatibility and cell adhesion properties were improved by gelatin. A further advantage was the steady release of growth factors that enhanced cell growth [77].

In summary, gelatin is a biomaterial that remains very present today, as it has been proven to be part of promising hydrogels such as self-healing, adhesive or conductive hydrogels. This biomaterial can be implanted into self-healing hydrogels since it provides hydrogels with a proper structure with which to design dynamic hydrogels. Altogether, its complex structure may help to achieve complex adhesive hydrogels that attach to tissues and ensure regeneration. Finally, gelatin may support conductive systems with cell improvement properties such as biocompatibility or cell adhesion.

### 4.2. Gelatin in Drug Delivery Systems

Since gelatin can degrade in the presence of the body’s enzymes, it allows for the release of biological agents over time. Notably, depending on the subdued treatment, gelatin will be positively or negatively charged, resulting in interactions between electrically charged compounds that lead to a controlled delivery [79]. As a result, this biomaterial has been widely employed in different drug delivery systems including microparticles, scaffolds, and more recently, nanofibers [80,81,82].

#### 4.2.1. Tissue Regeneration

Gelatin-based drug delivery systems for tissue regeneration date back to the 2000s. These constructs were simple and yet effective hydrogels that allowed for the controlled release of growth factors such as BMP-2 or TGF-β1 for bone regeneration [24,83,84,85]. Subsequently, these biomaterial-derivative platforms were widely employed for the renewal of a myriad of tissues such as myocardial, nerve or wounds because of gelatin’s ability to release diverse biological elements [77,86,87,88]. Nonetheless, in the search to find a synergistic effect, a forefront strategy relies on combining different therapeutic agents in the same delivery system (known as dual delivery platforms) [89]. This technique enables better mimicking of living conditions and the opportunity to interact with more biologically active molecules.

##### Gelatin-Based Microparticles

**Figure 6:**
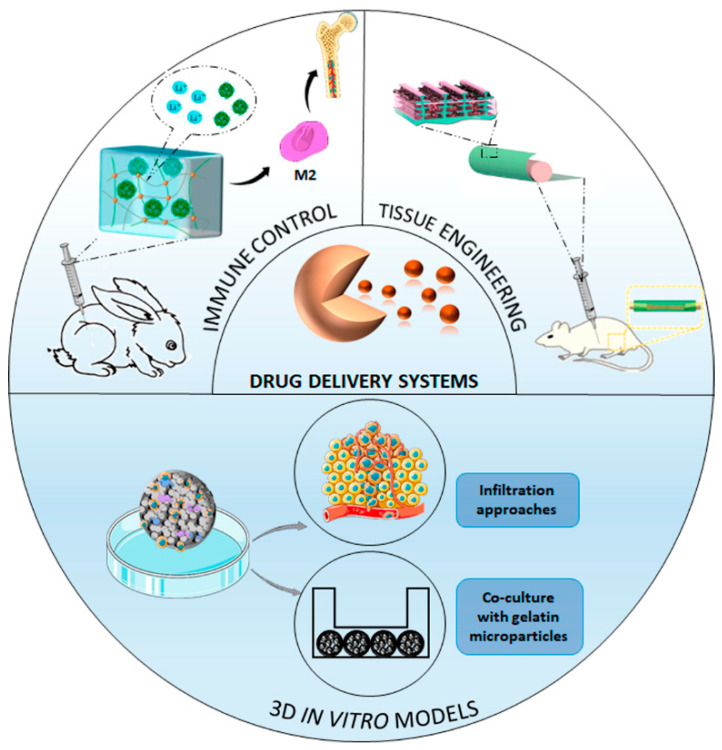
Representative graphic of gelatin-based drug delivery systems. Adapted with permission from Hu Y et al. [77]. ASC Nano. Copyright 2022, American Chemical Society; reprinted from Chemical Engineering Journal, 435, Li D et al. [84]. Osteoimmunomodulatory injectable Lithium-Heparin hydrogel with Microspheres/TGF-β1 delivery promotes M2 macrophage polarization and osteogenesis for guided bone regeneration, 134991, copyright (2022), with permission from Elsevier.

Microparticles have been demonstrated to be effective drug carriers, either to enhance tissue regeneration or to emulate in vitro performance [80] (Figure 6). Within this framework, the latest technologies have permitted a blending of dual liberation systems within injectable hydrogels [72,83,90]. Mitsui and coworkers recently designed a gelatin-based injectable hydrogel with embedded gelatin microspheres loaded with growth factors. These microparticles were responsible for releasing growth factors into the injectable hydrogel in order to promote cell growth and differentiation of the cells embedded there, which otherwise might have been reduced. Such a combination endorses taking advantage of the benefits of growth factors (such as cell proliferation or differentiation capacity) in a synergistic approach to guarantee the survival of the injected cells. In addition, the ability of the outer hydrogel to degrade in the presence of the enzyme collagenase helped to control the release of cells at the injured site [90].

##### Gelatin-Based Nanofibers

On the lookout for personalized medicine, nanofibers happen to be a candidate structure, since they bear a resemblance to an extracellular matrix [82,91]. Therefore, these particles have been part of a myriad of systems, ranging from drug delivery systems to scaffolding.

In this context, gelatin nanofibers have been employed in tissues such as tendon, cartilage and skin. Overall, supplementing scaffolding systems with gelatin-based nanofibers has addressed the lack of cell attraction and differentiation in comparison to other biomaterials. Likewise, it also serves as a platform for releasing biologically active agents (anti-inflammatory, antioxidants) that promote tissue regeneration [92,93,94,95,96]. In this sense, Nazarnezhad et al. developed a gelatin-based nanofiber skin substitute and it proved to be beneficial for re-epithelialization. The system showed prolonged degradation for up to 28 days and no cell toxicity. The addition of biological agents—blood derivatives rich in growth factors—increased cell viability and proved to provide a protective response against bacteria, which may help to prevent infections [94]. Similarly, in another recent study, gelatin-based nanofibers incorporated antioxidant agents to promote wound healing. As nanofibers mimic ECM structure, the complex demonstrated in vitro its capacity for cell adhesion and proliferation. Together with this, in vivo collagen formation was increased in the test group, which might be attributed to nanofiber’s drug delivery capacity [95]. In brief, gelatin-based nanofibers demonstrated in vitro ability to be considered as a scaffolding system as well as a drug delivery device. However, increased efforts are required to achieve efficient crosslinking methods for nanofibers, especially because conventional crosslinking methods applied to bulk gelatin materials would not be good enough to crosslink nanofibers with a higher surface area and consequently larger amounts of water, which may require pretreatment steps or crosslinking in a vacuum chamber [97].

#### 4.2.2. Immune Control for Tissue Engineering

Some recent studies have focused on analyzing the role of the immune system and inflammation in tissue regeneration, particularly, the regulation of macrophages [98,99]. A noteworthy study engineered BMP-2 loaded gelatin microspheres for bone regeneration. These systems responded to degradation enzymes expressed by M1 macrophages. Results show that microspheres were degraded over time and simultaneously BMP-2 was released to healing bone. This approach may be a possible strategy to control the release of growth factors, especially in the inflammatory phase of tissue regeneration [100].

The merging of the latest technologies using gelatin to build smart drug delivery systems has allowed a further step: to monitor migration from M1 to M2, bringing about a more detailed examination of the inflammatory process. For example, Yoshimoto et al. designed gelatin composite nanospheres as delivery systems, which allowed for the imaging of macrophage miRNA in the inflammatory process. The systems were complemented with imaging agents (molecular beacons) to detect miRNA in M1 macrophages, as it emits fluorescence without degrading the cell. The constructs were also immobilized by antibodies to be internalized in macrophages exclusively. The results showed that the nanospheres could be introduced into M1s and degraded over time, releasing fluorophores that underwent structural changes after interacting with miRNA. The latter enabled the detection of the proinflammatory phase of macrophages. This method represents a promising advance in better controlling the inflammation mechanism in tissue regeneration as it would permit at any time a vision of these immune system cells [101].

#### 4.2.3. In Vitro 3D Tissue Engineering

Despite the fact that gelatin has therapeutic properties for tissue regeneration or drug delivery, it also plays an important role in drug research. Specifically, since it has the ability to create 3D porous structures in which cells can grow, this biomaterial can imitate in vivo microenvironment conditions. In fact, gelatin has been used to culture different cancer and stromal cells [80,102,103].

Recent progress in this field has enabled the use of gelatin-based systems for drug-delivery purposes in in vitro 3D environments [104,105,106]. In this regard, Nii and co-workers designed chemically crosslinked, gelatin-based microspheres loaded with adenosine or Pifithrin-α drugs. These systems were embedded within tumor-cell aggregates. On the one hand, in both studies gelatin microspheres were shown to serve as a platform to enhance the long-term cell viability of the aggregates, given their ability to supply oxygen and nutrients [104,105]. On the other hand, the intricate 3D coculture of the tumor environment was further simulated by the controlled drug release. The constant release of adenosine resulted in the activation of tumor-associated macrophages, whereas that of Pifithrin-α proved to be effective in activating cancer-associated fibroblasts [104,105].

In short, gelatin may serve as an effective platform to deliver biological factors in culture systems, which is a step towards the design of more accurate 3D in vitro tumor-like models and it paves the way for investigating the performance of a wide range of cancer cells in the future.

### 4.3. Gelatin as Bioink for 3D Printing

As mentioned above, 3D printing has paved the way towards clinical use by designing high-precision and sophisticated systems, alongside high cost-effectiveness. Since gelatin is able to crosslink in situ as well as to provide biologically suitable properties (ability to promote cell adhesion, proliferation and differentiation), gelatin-based inks or its derivatives (for instance, GelMA) have been extensively exploited in several tissues such as bone, skin and cornea [107,108,109,110].

In the field of bone regeneration, Pu X and colleagues have recently designed a hydrogel derived from a gelatin-based bioink—composed of methacrylated gelatin and 80% hydroxyapatite—to insert in a rigid net. The high content of an inorganic component demonstrated good printability and improved the mechanical properties of the natural polymer, obtaining high rates of osteoconductivity. The printable hydrogel, in comparison to empty scaffolds, also prolonged degradation time and demonstrated its ability to promote cell adhesion, proliferation and differentiation in vitro as well as to regenerate bone tissue in vivo by attracting endogenous stem cells and creating vascular constructs [108]. Gelatin-derived ink also permits the integration of patient-derived stimulating compounds such as platelet-rich plasma or platelet-rich growth factors [109,111]. The addition of stimulating agents to bioinks may accelerate the regeneration process, since it provokes growths factor release and thus attracts more cells.

Gelatin-based ink has been exploited to regenerate cornea tissue. He B and coworkers designed a GelMA derivative ink. As the latter is photocrosslinkable and attracts cells, the 3D construct showed good cell attachment and differentiation. In vitro cell viability was not reduced either after seeding cells onto the surface of hydrogel or after printing cells within the gelatin-based ink. In addition, animal tests proved that 3D hydrogel was able to regenerate the cornea by stromal generation and re-epithelization [110].

As this natural polymer provides a suitable environment for cell growth, inks composed of such material have been employed to design several tissue-like systems or models (tumor, skin, muscle) [112,113,114]. A noteworthy example developed a heterogeneous tumor system based on a composite ink consisting of gelatin, alginate and cellulose. The construction demonstrated that the gelatin-based ink was suitable for printing various cell types within different shapes of the 3D system in vitro. Specifically, alginate and gelatin were responsible for biocompatibility and adhesion, respectively, while cellulose provided mechanical strength [114].

In summary, gelatin-based bioinks provide 3D systems with the potential to attract, attach and differentiate cells, resulting in complex structures capable of either promoting damaged tissue regeneration or designing cellular models.

### 4.4. Gelatin as Theranostic Agent

Theragnosis concentrates on finding materials that can be used to diagnose diseases while applying a therapy [115]. In this case, gelatin has shown that it can be part of different systems, some of which are focused on finding early markers for an early diagnosis, but others have proven to be effective for disease monitoring or evaluation.

One exciting area of research involves cancer therapy. Recently, gelatin has been blended with several signal-emitting materials (such as inorganic particles), to obtain an imaging of the disease course [116]. In this regard, Yadah et al. designed a blend of gelatin and inorganic particles. Therapy consisted of hepatoma ablation via NIR radiation, and imaging of the complex was obtained by functionalizing the system surface with indocyanine green, as it emitted optical signals upon stimulation. In this system, gelatin improves biocompatibility and biodegradability properties as the system is degraded in the presence of matrix metalloproteinases (abundant enzymes in the tumor environment), preventing the accumulation of inorganic particles in the body, which may become toxic in the long term [116].

Moreover, gelatin has also exhibited a role in cancer biomarker detection platforms. Recent studies have designed gelatin-containing systems that permit capturing blood circulating tumor cells (an early cancer biomarker). Entrapped cells, afterwards, are released steadily from the platform owing to the ability of gelatin to respond to physical stimuli (such as temperature). This gelatin-based platform allows for cancer cell isolation and posterior analysis as well as ensuring the encapsulated cells’ viability at all times, as a result of the biocompatibility provided by this biomaterial [117,118]. This novel system that traps cells and allows analyzing them in detail is a significant breakthrough in personalized medicine, as it would allow accurate diagnosis together with specific treatments for each case of tumor.

The field has branched out into other areas in order to detect biomarkers of certain diseases, such as psoriasis. Qiao et al. designed a gelatin-based microneedle patch to detect psoriasis-related RNA placed in the interstitial fluid. The study concluded that these systems could effectively detect biomarkers through a minimally invasive and automated approach. This forefront procedure may allow early diagnosis as well as imminent treatment in several diseases in the future [119]. Finally, the evaluation of the curing process is the focus in multiple systems. Gelatin has been involved in numerous devices that are capable of monitoring the course of the disease [76,120]. Mainly, these systems are designed to trace the wound healing process. In this line, Zheng et al. designed a gelatin-derived system able to respond to electrical stimuli, resulting in accelerated tissue regeneration, while at the same time, as it was able to control all the movements of the skin, the gelatin-based system improved the repair process [120].

## 5. Conclusions

Gelatin has been a widely explored biomaterial in multiple systems (hydrogels, scaffolds, drug delivery systems) and for extended applications in the field of tissue engineering. Despite this extensive use, several limitations remain to be addressed. For instance, the need to crosslink their chains forces them to be used in combination with other compounds, which, in turn, hinders the handling. Nonetheless, new technologies together with advanced techniques are providing interesting and emerging opportunities for this biomaterial, making gelatin a very versatile tool. First, simply being able to use this biomaterial suitable for 3D equipment has simplified its manipulation and has expanded its use to more complex tissues such as nerve or adipose. In parallel, functionalization has provided a synergistic effect in gelatin-based hydrogels, especially those designed by 3D printers, since it allows the material to be adapted to the injured tissue. These developments are opening new windows for gelatin in terms of therapeutic applications. Second, systems that are currently in the spotlight (drug delivery systems, self-healing, conductive or adhesive hydrogels) have taken advantage of gelatin’s characteristics as a means to improve their properties. A novel approach has also advocated the use of gelatin as a material to investigate new drugs or new therapeutic pathways in vitro. The latter may be useful in future trends that are shifting towards a new era that will focus not only on therapy, but also on disease diagnosis. In brief, gelatin’s potential to adapt to different environments, along with the timing, makes it a promising biomaterial for future therapeutic and theranostic approaches.

## Data Availability

Not applicable.
